# Supplementary material for: Hydroscapes: A Useful Metric for Distinguishing Iso-/Anisohydric Behavior in Almond Cultivars
Source: Plants (Basel). 2021 Jun 19;10(6):1249. doi: 10.3390/plants10061249 (PMC8233807; doi:10.3390/plants10061249)
Supplement: Supplementary file 1 [file plants-10-01249-s001.zip › plants-1194794-supplementary.pdf]

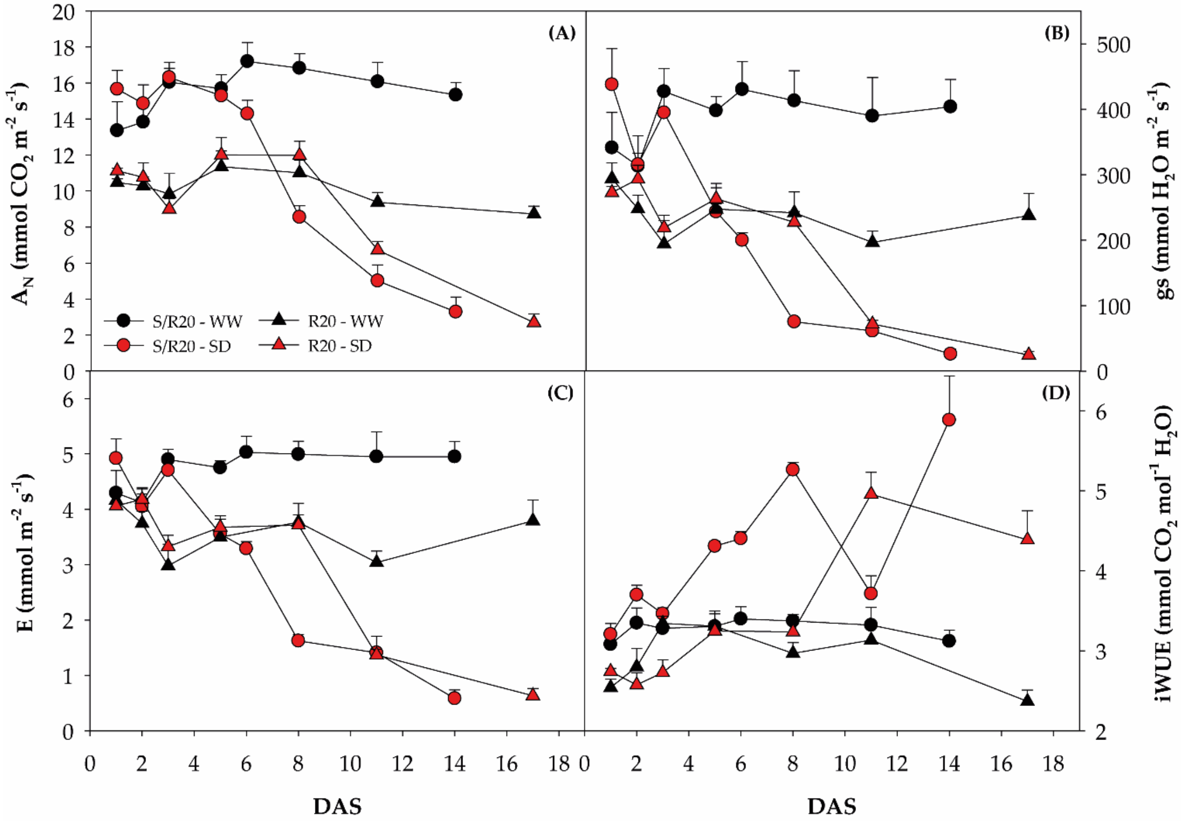

Figure S1. Evolution of net photosynthesis (A_N) (A), stomatal conductance (gs) (B), transpiration rate (E) (C) and intrinsic water use efficiency (iWUE) (D) during substrate desiccation experiment for S/R20 (circles) and R20 (triangles) cultivars under well-watered condition (WW, symbols in black) and substrate desiccation treatment (SD, symbols in red). Symbols indicate means + standard error. DAS: days after stress imposition.
